# Epidemiologic Features and Prognostic Factors of Coronary Artery Lesions Associated With Kawasaki Disease Based on a 13-Year Cohort of Consecutive Cases Identified by Complete Enumeration Surveys in Wakayama, Japan

**DOI:** 10.2188/jea.JE20140018

**Published:** 2014-09-05

**Authors:** Naomi Kitano, Hiroyuki Suzuki, Takashi Takeuchi, Tomohiro Suenaga, Nobuyuki Kakimoto, Shoichi Shibuta, Norishige Yoshikawa, Tatsuya Takeshita

**Affiliations:** 1 Department of Public Health, School of Medicine, Wakayama Medical University, Wakayama, Japan; 1 和歌山県立医科大学医学部公衆衛生学講座; 2 Department of Pediatrics, School of Medicine, Wakayama Medical University, Wakayama, Japan; 2 和歌山県立医科大学医学部小児科学講座; 3 Department of Pediatrics, Social Insurance Kinan Hospital, Wakayama, Japan; 3 社会保険紀南病院小児科

**Keywords:** coronary artery aneurysm, mucocutaneous lymph node syndrome, Kawasaki disease, epidemiology

## Abstract

**Background:**

To clarify the contribution of patient age to the development of coronary artery lesions (CALs) associated with Kawasaki disease (KD), epidemiologic features and prognostic factors were investigated using hospital-based complete enumeration surveys in a specific area.

**Methods:**

Consecutive KD cases identified between October 1999 and September 2012 in Wakayama Prefecture, Japan, were analyzed. The primary outcome measure was the presence/absence of CALs (giant aneurysm, mid- or small-sized aneurysm, and dilatation) on echocardiography 1 month after disease onset. Demographics and medical treatment factors were compared between the patients with and without CALs. Odds ratios (ORs) and 95% confidence intervals (CIs) of explanatory variables (age, gender, and factors related to high-dose intravenous immunoglobulin treatment) for the development of CALs were determined.

**Results:**

The median age of the 1415 patients (796 males, 619 females) was 25 months after excluding 2 children of foreign residents; 2.2% of the patients had a past history of KD, and 1.8% showed incomplete presentation. CALs were observed in 3.3% (4.0% of males, 2.3% of females; *P* = 0.080). The ORs of CALs among patients <11 months old (3.0, 95% CI 1.4–6.6) and those >48 months old (3.1, 95% CI 1.5–6.6) were significantly higher than values in 11- to 48-month-olds.

**Conclusions:**

The effect of patient age on the development of CALs was found to be U-shaped, with the bottom at ages 11 to 48 months. This finding was based on a 13-year cohort of consecutive KD cases in a specific area with little selection bias and is consistent with previously reported results.

## Abstract (ja)

**【目的】:**

本研究の目的は、日本の特定地域における全病院から回答を得た悉皆性のある調査結果を用いて、川崎病の臨床疫学的特徴および冠動脈病変発生の予測因子、特に罹患年齢を明らかにすることである。

**【方法】:**

1999年10月1日から2012年9月30日に和歌山県内で発症した川崎病症例を研究対象とした。本研究の主評価項目は、発症後1か月時点の冠動脈病変（巨大瘤、中・小瘤、拡大）発生の有無であり、経胸壁二次元心臓超音波検査所見を用いて評価した。冠動脈病変発生の有無により、対象の属性と治療内容を比較した。その後、多変量解析により、冠動脈病変発生に対する、罹患年齢、性別、免疫グロブリン大量療法の投与法・開始病日・追加投与のオッズ比と95％信頼区間を求めた。

**【結果】:**

研究対象期間に1417例の川崎病発生報告があった。両親のいずれかが外国人であった2例を除いた1415例（男796、女619）を解析対象とした。罹患年齢の中央値は25か月であった。再発例は2.2％で、不全型と診断されたのは1.8％であった。発症後1か月時点で、3.3％（男4.0％、女2.3％；*P*=0.080）に冠動脈病変発生を認めた。冠動脈病変発生のオッズ比は、罹患年齢11-48か月（第2-4五分位）を基準とした場合、10か月以下で3.0（95％信頼区間1.4-6.6）、49か月以上で3.1（1.5-6.6）と、いずれも有意に高値であった。

**【結論】:**

川崎病による冠動脈病変は、低年齢層と高年齢層で発生割合が高く、11-48か月を底とするU字型を示した。本研究は、日本の特定地域で13年間に発生した連続症例に基づくものであり、選択バイアスの影響がほとんどないことが特徴である。また、得られた結果は、川崎病全国調査による既報と一致するものであった。

## INTRODUCTION

Kawasaki disease (KD) is an acute febrile disease that predominantly affects infants and young children. KD was originally reported by the Japanese pediatrician Tomisaku Kawasaki in 1967.^1^^,^^2^ The etiology or responsible pathogen remains unknown. The diagnostic criteria for KD consist of six principal symptoms (prolonged fever lasting ≥5 days; bilateral nonpurulent conjunctival injection; erythema of the oral mucosa, lips, and tongue; polymorphous skin rash; erythematous indurations of palm and soles; and nonpurulent cervical lymphadenopathy).^3^^,^^4^ KD is markedly more prevalent in Asian countries, such as Japan (239.6 per 100 000 children less than 5 years old)^5^ and Korea (127.7 per 100 000 children less than 5 years old),^6^ than in Western countries.^7^ The epidemiologic characteristics of KD obtained based on nationwide surveys in Japan conducted almost every other year from 1970 indicate that seasonality, the occurrence of community outbreaks with a wave-like geographic spread, and time-space relationships suggest a relationship between KD and an infectious agent or agents.^8^^–^^11^ Higher rates of KD occur in siblings of index cases and twins, which suggests a possible role for genetic predisposition or for common exposure to an etiological agent or agents in the environment.^12^^,^^13^

Today, KD is a leading cause of acquired heart disease in children in developed countries.^14^^,^^15^ Shortly after the initial report by Kawasaki, KD was found to lead to myocardial infarction or sudden death with thrombotic occlusion of coronary artery aneurysms.^16^^–^^19^ High-dose intravenous immunoglobulin (IVIG) treatment against KD reduced the prevalence of the coronary artery complications,^20^^,^^21^ and the administration of IVIG in a single-infusion regimen of 2 g/kg was found to be more effective than a regimen of 400 mg/kg per day for 4 consecutive days.^22^ According to the nationwide surveys of KD in Japan, the prevalence of cardiac sequelae, defined as presence of dilatation of a coronary artery (including aneurysm), stenosis (including occlusion), and myocardial infarction or valvular lesion 1 month after disease onset,^19^ decreased from 18.0% in 1984 to 3.0% in the 2009–2010 survey.^5^

Various risk factors for cardiac sequelae of KD have been reported: male gender,^23^^–^^26^ infants,^23^^,^^27^^–^^29^ older children,^23^^,^^29^^,^^30^ recurrent cases,^31^ late IVIG treatment,^32^ and refractoriness to initial IVIG treatment.^28^^,^^31^^,^^33^^–^^36^ In the studies that reported that patient age was a risk factor for coronary artery damage in KD, the authors discussed the late start of initial IVIG treatment resulting from a late diagnosis and/or incomplete presentation, as part of their interpretation of the contribution of patient age.^29^^,^^30^

In the present study, to clarify the contribution of patient age to coronary artery lesions (CALs) associated with KD, the latest 13 years of consecutive cases of KD in a specific area were reviewed, and the prognostic factors for the development of CALs were examined, focusing on age, gender, and the interaction between age and gender.

## METHODS

This study protocol was approved by the ethics committee of Wakayama Medical University (Reference No. 794).

### Wakayama Kawasaki disease Study group (WKS) surveys

The study was performed in Wakayama Prefecture, Japan. Based on 2010 census data of this 4726-km^2^ area, there were 137 677 residents under 15 years of age, including 37 249 residents under 5 years of age (19 081 males, 18 168 females).^37^ In Wakayama Prefecture, a multicenter clinical and research network for KD, the Wakayama Kawasaki disease Study group (WKS), conducts hospital-based surveys of KD every October at all hospitals with a pediatric department. In these surveys, pediatricians are asked to review the medical records and report all patients with KD during the prior one-year period. The valid response rate of each survey has been 100%, and it is thus presumed that all KD cases diagnosed in Wakayama Prefecture are reported every year. The WKS network also includes the southernmost hospital with a pediatric department in Osaka Prefecture, and whether patients with KD living in Wakayama Prefecture admitted to the hospital crossed the prefecture’s northern border was examined. To the best of our knowledge, considering both the geographic characteristics and the healthcare situation not only in Wakayama Prefecture but also in the surrounding prefectures, these WKS surveys can be considered to represent all cases of KD in the study area.

The information obtained in the WKS surveys was as follows: age, gender, date of disease onset, history of KD (recurrence), diagnostic categories, use of oral aspirin therapy, use of IVIG treatment, date and regimen of initial IVIG treatment, administration of additional IVIG, and CAL findings.

### Study patients

In the present study, consecutive KD cases identified during a 13-year period (between October 1999 and September 2012) from the anonymous dataset composed of the annual surveys conducted by the WKS were reviewed. If one or both parents were foreign residents of Japan, that patient was excluded.

The diagnosis of KD was made on the basis of the criteria for the 4th or 5th (since 2002) Diagnostic Guidelines established by the Japan Kawasaki Disease Research Committee,^3^ which were used in the nationwide surveys in Japan. Two diagnostic categories are defined based on the number of principal symptoms observed: ‘complete presentation’ cases, diagnosed based on at least five items of the six principal symptoms or four of those items when coronary aneurysm or dilatation is seen on 2-dimensional echocardiography (2-DE); and ‘incomplete presentation’ cases, diagnosed based on four principal symptoms without coronary aneurysm or dilatation seen on 2-DE, or fewer principal symptoms with or without coronary aneurysm or dilatation seen on 2-DE.^3^ Refractoriness to initial IVIG treatment was defined as the presence or absence of additional IVIG treatment when the patient remained febrile (37.5°C or more) within 24 h after completion of the initial IVIG infusion.

### Evaluation of coronary artery lesions (CALs)

The primary outcome measure was the presence or absence of CALs (giant aneurysm, mid- or small-sized aneurysm, and dilatation). This outcome did not include transient dilatation of any coronary artery within 1 month after disease onset. The presence of CALs was measured using 2-DE performed at 1 month (about 30 days) after disease onset. The 2-DE findings were collected as multiple sets of measurements by pediatric cardiologists and/or well-trained pediatricians and interpreted by pediatric cardiologists. The present study used the Japanese Ministry of Health criteria published in 1984 as the “Report of the Subcommittee on the Standardization of Diagnostic Criteria and Reporting of Coronary Artery Lesions in Kawasaki Disease”,^38^ which have been adopted worldwide in epidemiologic studies of coronary artery complications in KD. A CAL was defined as follows: (1) lumen diameter (inside to inside) at least 3 mm in a child <5 years old or at least 4 mm in a child ≥5 years; (2) lumen diameter of a segment at least 1.5 times as large as that of an adjacent segment; or (3) clearly irregular lumen. A lumen diameter of any coronary segment ≥5 mm was defined as an aneurysm, and an aneurysm with a diameter measuring ≥8 mm was defined as a giant aneurysm.^38^

### Statistical analyses

The distributions of each variable in the study population, including demographics, were analyzed descriptively, and the data are shown as medians (interquartile ranges) for interval variables and numbers (percentages) for categorical variables. The patient demographics were compared among the four initial IVIG treatment regimens by chi-square test or analysis of variance. The patients’ demographics and medical treatment factors were compared between two groups: patients with or without one or more CALs at 1 month after disease onset. Differences were tested using the Mann-Whitney *U* test for interval variables or Fisher’s exact test for categorical variables.

The odds ratios (ORs) with 95% confidence intervals (CIs) of explanatory variables for the presence of CALs were determined by multiple logistic regression models adjusting for recurrence, incomplete presentation, and use of oral aspirin therapy. The explanatory variables considered in these models were age (three categories: less than 11 months old [first quintile], 11 to 48 months old [reference], and over 48 months old [last quintile]), gender (reference: female), initial IVIG treatment regimen (four categories: 2 g/kg for 24 h; 2 g/kg for 48 h; other regimens, including 2 g/kg for 5 days; and none [reference]), number of illness days when initial IVIG was started (three categories: less than 4 illness days, 4–7 illness days [reference], and more than 7 illness days), and the use of additional IVIG (reference: none). The computation of variance inflation factors showed no significant multicollinearity among the explanatory variables.

A two-tailed *P*-value of less than 0.05 was considered significant. SPSS, version 15.0 for Windows (Chicago, IL, USA), was used for all statistical analyses.

## RESULTS

Overall, 1417 patients with KD were reported during the 13-year study period. After the exclusion of two patients having at least one parent who was a foreign resident, the study population consisted of 1415 patients (796 males, 619 females; male/female ratio 1.29). The median age of the patients at disease onset was 25 months (range 1 to 122 months). Regarding the diagnostic categories, the percentage of incomplete presentation was 1.8% (26/1415), and there were no cases with less than three principal symptoms. The percentage of recurrent cases was 2.2% (31/1415).

The percentage who received IVIG treatment was 91.9% (1301/1415). The yearly distribution of the number of patients with KD is shown by the four categories of initial IVIG regimen (Figure). There were no significant differences across these four treatment regimen groups with respect to the patients’ gender, age at disease onset, or recurrence, but a significant difference in the groups’ proportions of incomplete presentation was observed (*P* < 0.001) (Table 1). Regarding the number of illness days when initial IVIG was started, 4.0% (57/1415) started at >7 illness days, which included 12 complete presentation cases starting at ≥10 illness days but none of the incomplete presentation cases.

**Figure.**
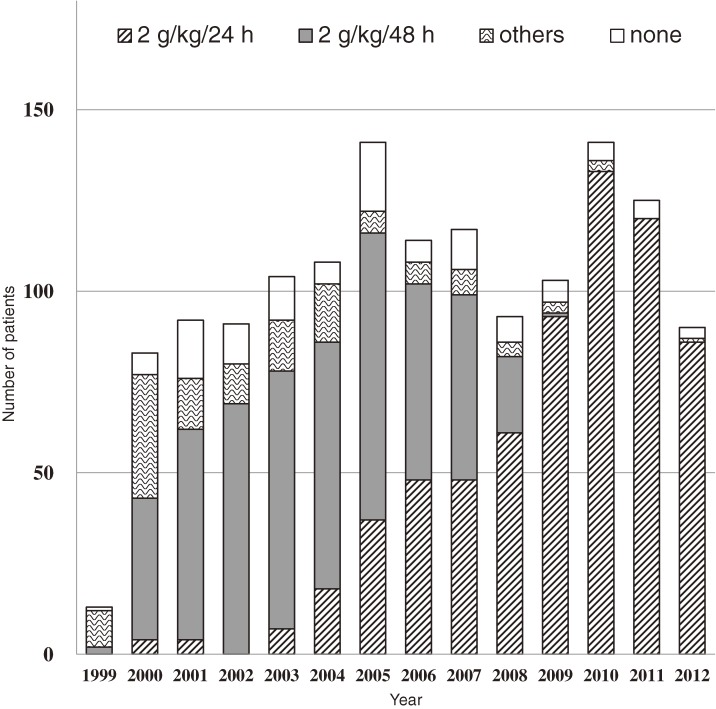
The distribution of the number of patients with KD from October 1999 to September 2012 by the four categories of initial IVIG treatment regimen (*n* = 1415).

**Table 1.** Characteristics of the 1415 patients with Kawasaki disease by the four categories of initial IVIG treatment regimen

	Initial IVIG treatment regimen				*P*-value^a^

	2 g/kg/24 h (*n* = 659)	2 g/kg/48 h (*n* = 513)	Others (*n* = 129)	None (*n* = 114)	
**Sex, male/female**	375/284	299/214	66/63	56/58	0.196
**Age, months; median** **[interquartile range]**	26 [13, 43]	25 [12, 43]	24 [13, 37.5]	24.5 [11, 47]	0.375
<11 months old, *n* (%)	132 (20.0)	100 (19.5)	26 (20.2)	21 (18.4)	0.979
11–48 months old, *n* (%)	400 (60.7)	305 (59.5)	86 (66.7)	68 (59.6)	0.510
>48 months old, *n* (%)	127 (19.3)	108 (21.1)	17 (13.2)	25 (21.9)	0.212
**Past history of KD**					
Recurrence, *n* (%)	13 (2.0)	13 (2.5)	3 (2.3)	2 (1.8)	0.910
**Diagnostic categories**					
Incomplete presentation, *n* (%)	4 (0.6)	3 (0.6)	4 (3.1)	15 (13.2)	<0.001

^a^*P*-values were based on chi-square test or analysis of variance.

The percentages of patients with CALs at 1 month after disease onset were 3.3% for the entire patient population (46/1415), 4.0% (32/796) for males, and 2.3% (14/619) for females (*P* = 0.080). Two males and four females had giant coronary aneurysms (0.42% [6/1415]). Table 2 shows the comparison of variables between the groups of patients with and without CALs. The proportion of patients with CALs was significantly lower in the group of 11- to 48-month-olds compared to the other age categories: 2.0% (17/859) and 5.2% (29/556), respectively (*P* = 0.001). There were no significant differences in the proportions of patients with CALs across the four categories of initial IVIG regimen. The number of illness days when initial IVIG was started was 2 to 6 in 97.6% (41/42) of patients with CALs and 23 in 1 complete presentation case; number of illness days was significantly fewer in patients with CALs than in those without CALs (*P* = 0.002). Regarding refractoriness to initial IVIG treatment, the overall percentage requiring additional IVIG was 18.0% (254/1415), and there was a significant difference in the percentage between the groups with (71.7% [33/46]) and without (16.1% [221/1369]; *P* < 0.001) CALs.

**Table 2.** Characteristics of study participants with and without coronary artery lesions (CALs) 1 month after disease onset

	Total	CALs (+) (*n* = 46)	CALs (−) (*n* = 1369)	*P*-value^a^
**Sex, male/female**	796/619	32/14	764/605	0.070
**Age, months (median [interquartile range])**	25 [12, 42]	33.5 [9, 54.8]	25 [12, 42]	0.396
<11 months old, *n* (%)	279 (19.7)	13 (28.3)	266 (19.4)	0.136
11–48 months old, *n* (%)	859 (60.7)	17 (37.0)	842 (61.5)	0.001
>48 months old, *n* (%)	277 (19.6)	16 (34.8)	261 (19.1)	0.013
**Recurrence, *n* (%)**	31 (2.2)	0 (0)	31 (2.3)	0.622
**Diagnostic categories**				
Incomplete presentation, *n* (%)	26 (1.8)	0 (0)	26 (1.9)	1.000
**Treatment**				
**Oral aspirin therapy, *n* (%)**	1393 (98.4)	46 (100)	1347 (98.4)	1.000
**Initial IVIG treatment, *n* (%)**	1301 (91.9)	42 (91.3)	1259 (92.0)	0.783
**Regimen of initial IVIG**^b^				
2 g/kg/24 h, *n* (%)	659 (46.6)	16 (34.8)	643 (47.0)	0.132
2 g/kg/48 h, *n* (%)	513 (36.3)	21 (45.7)	492 (35.9)	0.212
Others, *n* (%)	129 (9.1)	5 (10.9)	124 (9.1)	0.604
**Illness days of initial IVIG (median [interquartile range])**^b^	5 [4, 6]	5 [4, 5]	5 [4, 6]	0.002
<4 days, *n* (%)	62 (4.4)	5 (10.9)	57 (4.2)	0.047
4–7 days, *n* (%)	1182 (83.5)	36 (78.3)	1146 (83.7)	0.314
>7 days, *n* (%)	57 (4.0)	1 (2.2)	56 (4.1)	1.000
**Additional IVIG treatment, *n* (%)**	254 (18.0)	33 (71.7)	221 (16.1)	<0.001

^a^*P*-values were obtained by Mann-Whitney *U* test or Fisher’s exact test between CALs (+) and CALs (−).

^b^CALs (+) (*n* = 42) and CALs (−) (*n* = 1259).

Patient age had a U-shaped effect on the development of CALs. The risk of the development of CALs was significantly higher in patients <11 months old and in those >48 months old than in the group between 11 and 48 months old (OR 3.0, 95% CI 1.4–6.6 and OR 3.1, 95% CI 1.5–6.6, respectively) (Table 3).

**Table 3.** Results of multiple logistic regression analysis of coronary artery lesions (CALs) 1 month after disease onset (*n* = 1415)

	Univariate			Multivariate		
	
	OR	(95% CI)	*P*-value	Adjusted OR^a^	(95% CI)	*P*-value
**Gender**						
male	1.81	(0.96–3.42)	0.068	1.45	(0.73, 2.87)	0.285
female	1.00	reference		1.00	reference	
**Age**						
<11 months old	1.63	(0.85–3.15)	0.142	2.99	(1.35, 6.58)	0.007
11–48 months old	1.00	reference		1.00	reference	
>48 months old	2.26	(1.22–4.22)	0.010	3.11	(1.47, 6.60)	0.003
**Regimen of initial IVIG treatment**						
2 g/kg/24 h	0.60	(0.33–1.12)	0.107	0.11	(0.03–0.40)	0.001
2 g/kg/48 h	1.50	(0.83–2.70)	0.180	0.24	(0.07–0.84)	0.025
Others	1.22	(0.48–3.16)	0.675	0.42	(0.09–1.87)	0.253
Absence	1.00	reference		1.00	reference	
**Illness days of initial IVIG**						
<4 days	2.81	(1.07–7.37)	0.036	2.84	(0.93–8.66)	0.067
4–7 days	1.00	reference		1.00	reference	
>7 days	0.52	(0.07–3.85)	0.523	0.78	(0.09–6.42)	0.816
**Additional IVIG treatment**						
presence	13.19	(6.83–25.5)	<0.001	19.14	(8.73–41.98)	<0.001
absence	1.00	reference		1.00	reference	

^a^Adjusted for recurrence, incomplete presentation, and use of oral aspirin therapy.

Regarding the initial IVIG regimen, compared to patients without IVIG, those with the 2 g/kg/24 h regimen and those with the 2 g/kg/48 h regimen were significantly less likely to have CALs (OR 0.11, 95% CI 0.03–0.40 and OR 0.24, 95% CI 0.07–0.84, respectively). Regarding refractoriness to initial IVIG treatment, patients with additional IVIG administration were significantly more likely to have CALs (OR 19.1, 95% CI 8.7–42.0) than those without additional IVIG administration.

In this study population, no significant association was seen between patient gender and the development of CALs. In the multiple logistic regression analyses stratified by age groups, no significant interaction between patient age and gender was observed (data not shown).

## DISCUSSION

The results of the present study demonstrate that patient age at the onset of KD played an important role in the development of CALs. The effect of patient age on the development of CALs, including giant aneurysms, mid- or small-sized aneurysms, and dilatation, was U-shaped; both patients less than 11 months old (infant group) and those over 48 months old (advanced age group) had a significantly higher risk of developing CALs than patients 11 to 48 months old at disease onset.

The present study has two strengths: (1) the study population consisted of consecutive cases identified by a 13-year series of hospital-based complete enumeration surveys in a specific area, and (2) data quality was managed by pediatric cardiologists. In WKS, the diagnostic categories (the inclusion criteria), decision-making related to IVIG treatment, and 2-DE findings (the primary outcome measure) were well-managed by pediatric cardiologists. In the present study, there were no patients with CALs who received a delayed diagnosis of KD. Further, the percentage of incomplete presentation in the WKS dataset was much lower than that reported from nationwide surveys in Japan, even though the annual incidences of KD in Wakayama Prefecture were continuously higher than those in Japan overall.^5^^,^^11^ Using data with little selection bias and only a small proportion of incomplete presentations, the present finding confirmed that both the infant group and the advanced age group were vulnerable to the development of CALs.

Three nested, matched case-control studies using the nationwide surveys of KD in Japan examined the effect of age, focusing on giant coronary aneurysms.^33^^–^^35^ To the best of our knowledge, this is the first study dealing not only with giant aneurysms but also with mid- or small-sized aneurysms and dilatations as the primary outcome measure. The finding of a U-shaped effect of patient age on the development of CALs suggests heterogeneity of causative agents or different reactions at different maturity stages of the immune and vascular systems associated with the pathogenesis of damage to coronary arteries in KD.

Several studies in Canada,^39^ Taiwan,^40^ and Korea^41^ have discussed the issue of the relationship between patient age and coronary artery complications in KD. In these reports, however, both the incidence of KD in children and the proportion of coronary artery complications differed among countries/areas or by race/ethnicity. There were also differences in not only the studies’ designs, including sampling method, definition of KD (eg whether fever persisting for 5 days or more was essential), and the validity of the evaluation of coronary arteries, but also in the health insurance system and consultation behavior, socioeconomic status, healthcare standards, and quality of medication, all of which should be considered when comparing the results.^42^^,^^43^

As protective factors against the development of CALs in KD, the initial IVIG treatment with regimens of 2 g/kg for 24 h and 2 g/kg for 48 h was found to reduce the risk of the development of CALs as evaluated by 2-DE at 1 month after disease onset. This finding strengthens the growing body of results from nationwide surveys in Japan, although there were some differences in regimen categories between the present and related studies.^33^^,^^34^ With respect to the association between the refractoriness to initial IVIG treatment and the presence of CALs, the present finding is also consistent with the results of nationwide surveys in Japan.^33^^–^^36^

The present results indicated the vulnerability of males to KD, but no significant gender difference in the development of CALs evaluated at 1 month after disease onset was observed. Hirose et al. reported that the male-female ratios of the cumulative incidence rates of KD-related cardiac sequelae were around 2.0 in all birth-year cohorts.^44^ We plan to investigate the effects of gender and the interaction between age and gender on the development of CALs in a larger cohort of patients with KD.

The present study has some limitations. First, the study patients were limited to a specific area. Consecutive cases over a 13-year period in 1 prefecture in Japan (similar to a state in the USA) were examined; thus, the findings obtained in the present study were based on the data from a study population consisting of Japanese patients with KD and cannot be directly applied to KD patients of different racial or ethnic backgrounds. However, the incidence of KD in Japan is extremely high, and the findings in the present study could be used as a representative or reference sample. Second, it is difficult to prove that the present study was based on all cases in the survey area. While the possibility of the presence of missed cases (eg transition between prefectures) remains, to the best of our knowledge, all patients with KD who lived in Wakayama Prefecture in the study period were reviewed. The need for a community-based registration system of patients with KD should be considered to resolve the etiology of KD and prevent acquired cardiovascular disease. Third, the quality management of the measurements of coronary artery diameters as outcome indicators should be mentioned. Ultrasonographic examinations through a transthoracic probe provide diagnostic images that are suboptimal. They have some weaknesses regarding reliability and validity, such as intra- and inter-individual variation among examiners. However, in the present study, the medical specialty of each examiner was deemed appropriate, and the interpretations of individual echocardiographic findings were prospectively managed by pediatric cardiologists, even though it was a multicenter study.

Regarding the definition of CALs in the present study, the development of CALs was evaluated using a dichotomous definition previously published in Japan by the Research Committee on Kawasaki Disease of the Ministry of Health, as in many prior studies.^5^^,^^6^^,^^17^^,^^23^^–^^26^^,^^30^^–^^36^^,^^38^^–^^40^^,^^43^^,^^44^ The diameters of coronary arteries were not adjusted by body surface area in the present study analyses, because, at the time the WKS surveys were started, the use of serial normalized coronary artery measurements (z-scores) calculated from the inner diameter of the coronary artery and body surface area had not been established in children.^45^ The risk factors for CALs determined by analyses of serial z-scores were reported to be similar to those determined using dichotomous definitions, particularly the Japanese Ministry of Health criteria of maximal luminal dimension.^46^ In epidemiologic studies, the validity or usefulness of z-scores of coronary arteries of patients with KD has not been established.

In conclusion, children aged both less than 11 months and over 48 months are at higher risk of developing CALs associated with KD than those aged 11–48 months. This U-shaped finding for patient age was from a 13-year cohort of Japanese patients with little selection bias in a specific area, nearly equivalent to a community-based complete enumeration study. The present finding is consistent with the results of previous studies assessing the risk of giant aneurysms in KD and suggests that the heterogeneous features of KD are due to different agents or an interaction between genetic and environmental factors modified by patient age in the development of CALs.

## ONLINE ONLY MATERIALS

Abstract in Japanese.
