# Supplementary material for: Epidemiologic Features and Prognostic Factors of Coronary Artery Lesions Associated With Kawasaki Disease Based on a 13-Year Cohort of Consecutive Cases Identified by Complete Enumeration Surveys in Wakayama, Japan
Source: J Epidemiol. 2014 Sep 5;24(5):427–34. doi: 10.2188/jea.JE20140018 (PMC4150015; doi:10.2188/jea.JE20140018)
Supplement: Abstract in Japanese. [file je-24-427-s001.pdf]

# 川崎病の疫学的特徴と冠動脈病変の予測因子 -和歌山県における13年間の悉皆的調査から

北野尚美[1]，鈴木啓之[2]，武内　崇[2]，末永智浩[2]，垣本信幸[2]，渋田昌一[3]，吉川徳茂[2]，
竹下達也[1]，和歌山川崎病研究会グループ

[1] 和歌山県立医科大学医学部公衆衛生学講座
[2] 和歌山県立医科大学医学部小児科学講座
[3] 社会保険紀南病院小児科

【目的】本研究の目的は、日本の特定地域における全病院から回答を得た悉皆性のある調査結果を用いて、川崎病の臨床疫学的特徴および冠動脈病変発生の予測因子、特に罹患年齢を明らかにすることである。
【方法】1999年10月1日から2012年9月30日に和歌山県内で発症した川崎病症例を研究対象とした。本研究の主評価項目は、発症後1か月時点の冠動脈病変（巨大瘤、中・小瘤、拡大）発生の有無であり、経胸壁二次元心臓超音波検査所見を用いて評価した。冠動脈病変発生の有無により、対象の属性と治療内容を比較した。その後、多変量解析により、冠動脈病変発生に対する、罹患年齢、性別、免疫グロブリン大量療法の投与法・開始病日・追加投与のオッズ比と95%信頼区間を求めた。
【結果】研究対象期間に1417例の川崎病発生報告があった。両親のいずれかが外国人であった2例を除いた1415例（男796、女619）を解析対象とした。罹患年齢の中央値は25か月であった。再発例は2.2%で、不全型と診断されたのは1.8%であった。発症後1か月時点で、3.3%（男4.0%、女2.3%；$P$=0.080）に冠動脈病変発生を認めた。冠動脈病変発生のオッズ比は、罹患年齢11-48か月（第2-4五分位）を基準とした場合、10か月以下で3.0（95%信頼区間1.4-6.6)、49か月以上で3.1（1.5-6.6）と、いずれも有意に高値であった。
【結論】川崎病による冠動脈病変は、低年齢層と高年齢層で発生割合が高く、11-48か月を底とするU字型を示した。本研究は、日本の特定地域で13年間に発生した連続症例に基づくものであり、選択バイアスの影響がほとんどないことが特徴である。また、得られた結果は、川崎病全国調査による既報と一致するものであった。

【キーワード】冠動脈瘤、川崎病、粘膜皮膚リンパ節症候群、MCLS、疫学
